# Screening in asymptomatic coronary artery disease: helpful, redundant or harmful?

**DOI:** 10.1007/s12471-014-0614-0

**Published:** 2014-10-18

**Authors:** E. E. van der Wall

**Affiliations:** Interuniversity Cardiology Institute of the Netherlands (ICIN), Netherlands Heart Institute (NHI), PO Box 19258, 3501 DG Utrecht, the Netherlands

Screening asymptomatic individuals remains the subject of intense debate in medicine. In the beginning of 2014 the use of screening for breast cancer in women was seriously questioned based on studies from Canada (Toronto) and the Netherlands (Leiden). It was shown that, particularly in women over 70 years, screening for breast cancer might even bring more harm than benefit in terms of over diagnosis and thus overtreatment. With respect to carotid artery disease, in September of this year the United States Preventive Services Task Force (USPSTF) published a recommendation to oppose the screening of the general adult population to detect asymptomatic carotid artery stenosis [1]. The Task Force found that ultrasound may produce false-positives resulting in angiography or even surgery, with the accompanying high risk of stroke, heart attack, or death. This recommendation may be important because it may influence insurance coverage.

How about screening individuals with asymptomatic coronary artery disease? In a review article from the Netherlands (Rotterdam) a systematic review of guidelines on imaging of asymptomatic coronary artery disease showed the light in 2011 [2]. It turned out that guideline on risk assessment by imaging of asymptomatic coronary artery disease contained conflicting recommendations. Out of the 14 guidelines that met the inclusion criteria, eight guidelines recommended against or found insufficient evidence for testing individuals with asymptomatic coronary artery disease. The authors suggested therefore that more research, including randomized controlled trials, is needed to evaluate the impact of imaging on clinical outcomes and costs.

Over the past years cardiac computed tomography (CT) has emerged as a screening tool - in addition to the proven use of major risk factors - to detect coronary artery disease in an early stage of its process [3–7]. Both calcium scoring and coronary CT angiography have been shown useful to identify coronary artery lesions. However, how does one manage the issue of using coronary CT as a screening test in asymptomatic individuals? According to the 2013 ESC Guidelines on Stable Angina Pectoris [8], both coronary calcium detection by CT and coronary CT angiography receive a Class III indication, meaning that there is ‘*evidence and/or general agreement that the procedure is not useful/effective and in some cases may be harmful*’. The inherent guideline recommendations were twofold: 1) coronary calcium detection by CT is not recommended to identify individuals with coronary artery disease, and 2) coronary CT angiography is not recommended as a screening test in asymptomatic individuals without clinical suspicion of coronary artery disease.

Apart from the lack of usefulness of applying CT in asymptomatic individuals one should always be aware of the risk of radiation exposure using ionising imaging modalities to detect patients with coronary artery disease. To that purpose, the American Heart Association (AHA) very recently issued a scientific statement on cardiac imaging, published online on 29 September 2014 in the journal Circulation [9]. The AHA scientific statement recommended that exposure to radiation should be part of the discussion on cardiac imaging for both referring and performing physicians. Physicians should be required to know which cardiac imaging tests use ionising radiation, understand the basics of exposure, and know the typical dose estimates for the most commonly used cardiac imaging procedures. In addition, they should counsel patients on the risks as well as on potential benefits so that patients can give truly informed consent. Consequently, before referring a patient for a cardiac imaging test, the AHA recommends that physicians address important questions such as 1) how will the test help diagnose or treat the cardiac problem, 2) are there alternative modalities not using radiation, 3) what are the levels of radiation exposure, 4) how will it affect the risk of cancer later in life, and 5) how does that compare with the risk from other common activities. These questions remain pertinent despite successful technological attempts to reduce radiation exposure, which holds in particular for cardiac CT.

To conclude, although screening for coronary artery disease -especially using advanced ionising cardiac imaging techniques [10] - may be helpful in patients with a intermediate to high probability of disease, it may be redundant in patients with a low prevalence of disease and even harmful in asymptomatic individuals.
